# The Role of Circadian Misalignment due to Insomnia, Lack of Sleep, and Shift Work in Increasing the Risk of Cardiac Diseases: A Systematic Review

**DOI:** 10.7759/cureus.6616

**Published:** 2020-01-09

**Authors:** Safeera Khan, Bilal Haider Malik, Deepti Gupta, Ian Rutkofsky

**Affiliations:** 1 Family Medicine, California Institute of Behavioral Neurosciences and Psychology, Fairfield, USA; 2 Internal Medicine, California Institute of Behavioral Neurosciences and Psychology, Fairfield, USA; 3 Reproductive Medicine, Saint Mary's Hospital, Manchester, GBR; 4 Psychiatry, California Institute of Behavioral Neurosciences and Psychology, Fairfield, USA

**Keywords:** circadian misalignment, shift work, insomnia, decreased sleep, cardiovascular risk

## Abstract

Around 121.5 million people suffer from cardiovascular diseases globally. The risk of cardiovascular diseases increases with advancing age in both genders. Circadian rhythm is responsible for a streamlined functioning of various body functions. Certain functions and hormones have their peak levels according to the biological day or night of circadian rhythm. Shift work and sleep disorders like obstructive sleep apnea can cause circadian misalignment that affects different metabolic, immunological, and cardiovascular functions, which ultimately increases the risk of cardiovascular diseases. We systematically searched the online database PubMed to find papers on randomized controlled trials (RCTs) from the past five years, evaluating the role of shift work and different sleep disorders in causing circadian misalignment and its effect on the risk of cardiovascular diseases. Fifty papers were shortlisted, and after the application of various inclusion and exclusion criteria, 18 papers were chosen; and then after a thorough analysis of the text, eight papers were selected for the review. All papers were evaluated for quality. Two papers focused on the effect of shift work on cardiovascular diseases, whereas five papers evaluated the role of sleep disorders on circadian rhythm and the risk of cardiovascular diseases. Shift work and sleep-related disorders were found to cause circadian misalignment, and it was found to be associated with an increase in the risk of cardiovascular diseases. Managing these disorders can help reduce the risk of cardiovascular diseases.

## Introduction and background

Around 121.5 million adults suffered from cardiovascular diseases in 2016, which makes up around 48% of the population of the US. Around 46% of the adult population in the US suffers from hypertension [1]. This incidence of cardiovascular diseases progressively increases in both genders with advancing age. According to 2016 data, cardiovascular diseases cause about 2,303 deaths in a day, with a person dying every thirty-eight seconds [1]. This high rate of morbidity and mortality can be curtailed with proper control of the risk factors. Along with main risk factors like hypertension, diabetes, obesity, and metabolic syndromes, some other factors like sleep disturbances and stress might play a significant role. The disruption of circadian rhythm is also considered a potential contributor to cardiovascular diseases.

Humans just like other mammals have an inbuilt clock that manages the timings of various body functions. We have a period of activity where these functions are at their peak and a period of inactivity when the functions are sluggish. This inner clock is called the circadian rhythm, which divides the 24 hours into a ‘biological day’ and a ‘biological night’ irrespective of the actual day or night [2]. This biological change also causes changes in body temperature, circulating cortisol and melatonin levels [3,4]. Suprachiasmatic nucleus and the circadian oscillators make up the circadian system. The suprachiasmatic nucleus, located in the hypothalamus, is the controlling center of the circadian system. Circadian oscillators, on the other hand, are present in many peripheral organs like the heart, pancreas and liver, which explains the cyclical activity or the difference in the performance of cardiovascular and metabolic functions at different times. These circadian oscillators generate a circadian rhythm of their own. Both of these synchronize with each other to perform cyclic functions [2]. This synchronization may be lost if the connection between the center and peripheral circadian controllers is disrupted. This circadian rhythm is in alignment with the external timings of the day. The biological day is aligned with the daytime or the light time, whereas the biological time is aligned with the night time or the dark period. This alignment is lost when the inner timings are at a different time than the actual time of the day and are not in sync with each other, and this is called circadian misalignment. This misalignment can occur because of jet lag, shift work, sleep disturbances like insomnia, obstructive sleep apnea, etc. Circadian misalignment may lead to different cardiovascular and metabolic health problems by impairing the biological processes like insulin sensitivity, immunity, blood pressure, and cardiac autonomic control, and may increase the risk of certain diseases [5].

Our focus is on the major cardiovascular risk factors, and there is a huge knowledge gap regarding sleep-related issues and circadian misalignment as risk factors for these cardiovascular diseases or metabolic diseases like diabetes, which in turn becomes a major risk factor for cardiac diseases.

This review will focus on assessing the effect of factors causing circadian misalignment, like shift work or sleep disturbances, on the risk of development of cardiac diseases. Understanding this role can help in the prevention of cardiovascular diseases by addressing these issues and may help to improve cardiac patient’s health.

## Review

Methods

**Figure 1:**
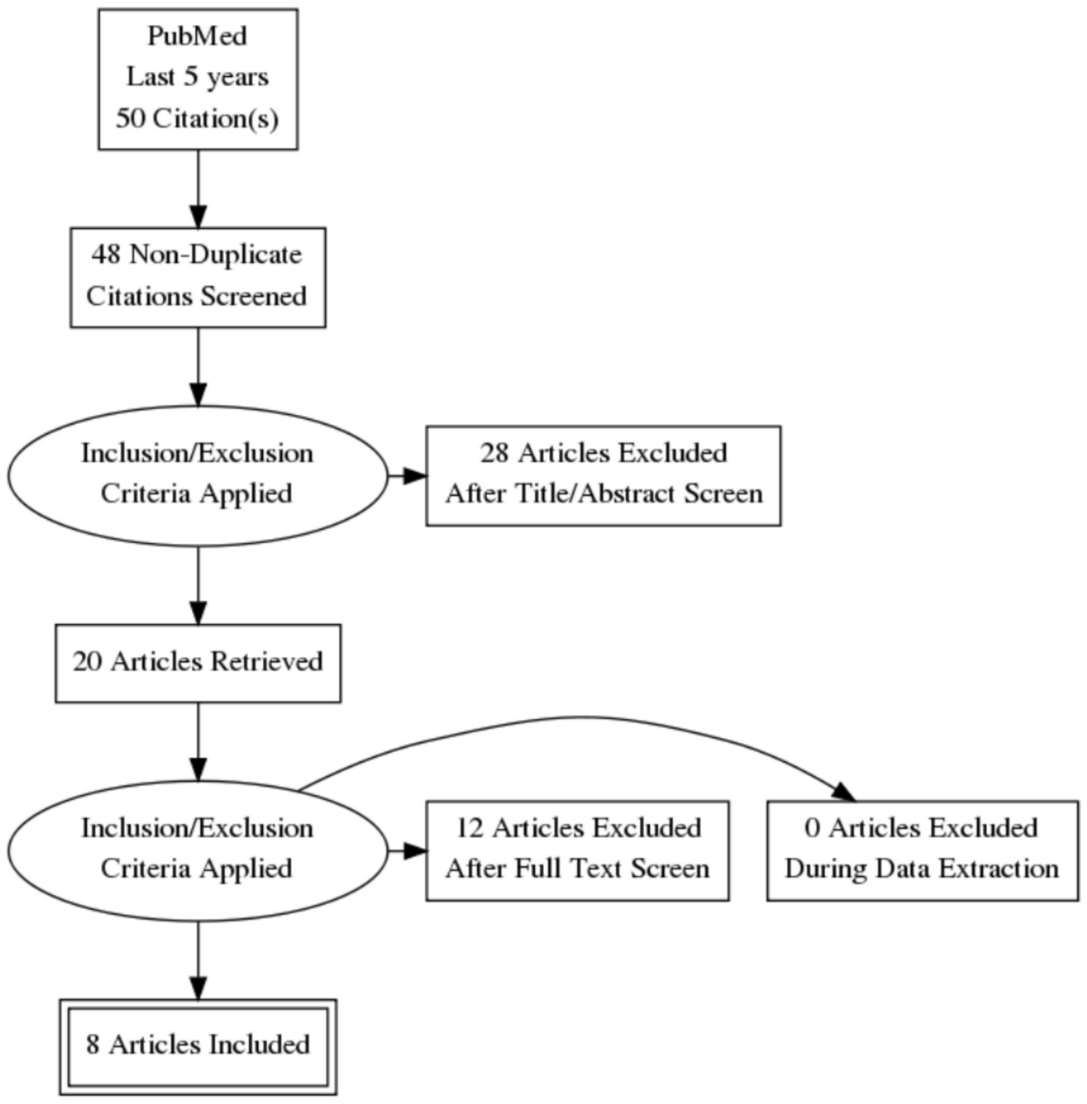
Prisma diagram showing the selection of studies

We searched the online database PubMed systematically for our data collection. We specifically searched for randomized controlled clinical trials assessing the association of circadian misalignment due to sleep disturbances or shift work with cardiac diseases. We used cardiovascular risk, shift work, insomnia, circadian misalignment, cardiac diseases, and arrhythmias as keywords, both alone and in combination to search for the published papers from the past five years. Fifty papers that were in the English language were extracted. Twenty papers were eventually shortlisted after removing abstract reviews and duplicate papers. Then inclusion and exclusion criteria were applied, which yielded 12 papers. A total of eight articles were finalized after the full-text review as shown in Figure 1. All the selected papers were peer-reviewed and were assessed for quality.

Inclusion and exclusion criteria

The papers in the English language from the past five years evaluating the association of circadian misalignment and cardiac diseases were selected. Only peer-reviewed papers were included. Review papers, unpublished studies, or research papers in other languages were excluded. Papers assessing metabolic or immune effects of circadian misalignment were also excluded.

Results

Out of these eight selected clinical trials, four of them assessed the role of shift work as a cause of circadian misalignment in cardiac diseases [6,7,8,9]. Five among them assessed the sleep-related issues like insomnia, obstructive sleep apnea and lack of sleep as a contributing factor in cardiac diseases [8,10,11,12,13]. One of the papers assessed all three of them as a potential contributing factor in cardiac diseases [7]. Table 1 shows the selected studies from the review [6-13].

**Table 1:** Description of selected studies from the review RCT: randomized controlled trial; ACS: acute coronary syndrome; PCI: percutaneous cardiac intervention; STEMI: ST-elevation myocardial infarction; CBT: cognitive behavioral therapy; TCC: Tai Chi CHIH; SS: sleep seminar

Sand year	Type of study	Purpose of study	Result/conclusion
Morris et al, 2015 [6]	RCT	Evaluated the impact of circadian misalignment in a real-life shift worker	Circadian misalignment causes a rise in inflammatory markers and blood pressure which are known risk factors of cardiac diseases
Barger et al, 2017 (SOLID-TIMI 52 trial) [7]	RCT	Sleep duration, sleep apnea, and shift work are independent risk factors of cardiovascular diseases	Patients having a shorter duration of sleep (less than six hours) had an increased risk of serious cardiovascular events, than those having longer sleep. Patients with obstructive sleep apnea had 12% more risk of cardiovascular events than patients with normal sleep. Overnight shift work also had a higher risk than those working in the day. They are the predictors of prognosis and serious events in patients with ACS
Jarrin et al, 2018 [8]	RCT	Assessed the role of sleep abnormalities and shift work on the development of heart failure due to loss of parasympathetic control	Insomnia patients having short sleep duration had reduced parasympathetic activity as compared to those with normal sleep duration insomnia. They also showed an increased imbalance between sympathetic and parasympathetic balance. Treating insomnia may reduce the risk of cardiovascular diseases
Dutheil et al, 2017 [9]	RCT	Compared the cardiac stress in Emergency Physicians during the night shift of fourteen hours with the 24-hour shift and to evaluate factors affecting cardiovascular events	Cardiac stress was double during a shift than in the regular days. There were multiple episodes of tachycardia during shifts with HR even up to 180 bpm. Tachycardia of more than 100 bpm was more in 24-hour shift and for a longer duration. The serious emergencies increased the duration and the number of episodes of tachycardia. Stress-induced more tachycardia having >100, 110, or 120 bpm
Kanno et al, 2016 [10]	RCT	Assessed the effect of insomnia on heart failure	The rate of serious cardiac events was significantly higher in persons with insomnia than those with normal sleep. The insomnia group was found to have high Rennin and Aldosterone levels and activity. Heart-failure patients with insomnia have activated the rennin-aldosterone system; therefore insomnia can be an independent predictor of serious cardiac events
McGrath et al, 2017 [11]	RCT	Determined the role of sleep intervention therapy in reducing blood pressure compared to risk factor education	The sleep intervention group showed improvements in sleep quality. The web-based sleep intervention showed improvement in sleep and psychosocial health but the blood pressure was not decreased for a short duration
Gheili et al, 2018 [12]	RCT	Compared the role of Melatonin and Oxazepam in the management of insomnia in cardiac patients following PCI	Melatonin was found to improve sleep quality more than Oxaxepam, it also improved the anxiety and other risk factors more than Oxazepam in patients with STEMI
Carroll et al, 2015 [13]	RCT	Determined the comparative efficacy of CBT, TCC, and SS to reduce multisystem biomarkers of disease risk in older adults with insomnia	Patients with a high risk of developing cardiovascular diseases reduced the risk with improved sleep and so the likelihood to be in the high-risk group was also decreased at 16 months. The risk of chronic diseases in older adults can be reduced by improving sleep quality as it can improve cardiovascular, metabolic, and inflammatory markers

Limitations

We only found three clinical trials assessing the effect of shift work on cardiac diseases from the past five years, and two of these did not evaluate the nature of the job in shift work or the stress involved during that shift that may exacerbate its effect on overall risk of cardiovascular diseases. Therefore, the actual increase in risk with shift work could not be fully evaluated.

Discussion

The inbuilt system or circadian rhythm is responsible for the streamlined functioning of the metabolic and immune systems. Cortisol release is associated with the circadian rhythm; it starts to rise a few hours after sleep and is at its peak in the early morning and in early awakening hours, helping the body transition from biological night to biological day. This circadian release is affected by the disturbed sleep; it increases in insomnia or decreased sleep. Similarly, shift work also disrupts this normal release pattern, causes fatigue, and affects the release of epinephrine and norepinephrine, which ultimately affects the heartbeat variability, blood pressure, and heart rate. Melatonin starts to increase after dark, reaches a peak by midnight, and then gradually decreases in the latter half. Rotating shift work causes abnormal melatonin levels [14]. Melatonin levels showed an association with cardiovascular events, and it was found to be decreased in patients with coronary artery diseases, and the more it was low, more was the risk of cardiovascular events like myocardial infarction (MI), showing that severity was inversely proportional to the levels of melatonin [15]. Certain cardiovascular variables change in a diurnal pattern with the circadian pattern, these include heart rate, blood pressure, and fibrinogen activity [16]; so does platelet activity, lipid metabolism, endothelial function, and vascular tone [17], which may partly explain why cardiovascular events occur mostly in the morning and follow a circadian rhythm [17]. Sheer at al. demonstrated the association of platelet activity with circadian rhythm [18], which has the highest activity in the early morning [19]. All these changes are occurring by the inner circadian rhythm; therefore, these peripheral oscillators themselves follow the circadian rhythm and so can be affected by circadian misalignment. Another factor predicting the risk of cardiovascular diseases is the level of inflammatory markers like interleukins [20].

Effect of Shift Work and Sleep Disorders

Many scientists have suggested that factors like shift work cause circadian misalignment by making the body work opposite to the physiological routine and contribute to cardiovascular events [21,22]. A meta-analysis showed that in adults, abnormal sleep duration of both less than seven hours or more than eight hours contributes to mortality and that reduced sleep is specifically associated with cardiovascular diseases and congenital heart diseases [1]. Shift work, which exposes a person to light at abnormal times, and sleep disturbances like insomnia, obstructive sleep apnea, and reduced or altered sleep pattern causes the loss of synchronization between normal circadian rhythm and the body functions which in turn increases the risk of cardiovascular diseases [23,24]. This circadian misalignment causes the production of hormones at times when they are not required in that much concentration and in reduced amount when they are needed the most, altering the flexibility of stress response; this may ultimately lead to glucocorticoid excess and thus cause its metabolic and cognitive consequences. These effects increase the risk of cardiac diseases.

An RCT by Moris et al. showed that the circadian misalignment can cause an increase in the blood pressure and the inflammatory markers like C- reactive protein (CRP), interleukin-6 (IL-6), and tumor necrotic factor (TNF), each of which can be an independent risk factor for cardiovascular diseases. These effects or the increase in these factors were enhanced with the increased duration of the circadian misalignment [6]. This blood pressure rise with the circadian misalignment was both in systolic and diastolic blood pressure [6]. Another multicenter study (SOLID-TIMI 52 trial) involving a long-term follow up of 13,026 patients shows the association of causes of circadian misalignment such as different sleep disorders like reduced sleep, obstructive sleep apnea, and shift work with the increased risk of cardiovascular disorders. Each of them is an independent risk factor and the effect is enhanced if a person has more than one risk factor [7]. Jarrin et al. conducted a study in which they reevaluated the data from two previous trials to analyze the link of two phenotypes of insomnia, one having normal sleep duration and one with short sleep duration, with cardiovascular outcomes. They found that the type of insomnia with short sleep duration reduced the parasympathetic activation, which ultimately caused a sympathovagal imbalance [8]. The heart rate and heart rate variation differed between the people having a normal duration of sleep and those having less than six hours of sleep over a short duration but not over a long period [8]. These changes caused by decreased parasympathetic activity can lead to tachycardia and raised blood pressure; it can also decrease insulin production and raise inflammation markers like cytokines, which are the exact changes depicting circadian misalignment. They can all contribute to developing cardiovascular morbidity [25,26]. If shift work is combined with stressful situation, it leads to even more frequent and persistent tachycardia, as was demonstrated by Dutheil et al. They monitored emergency physicians during a 14-shift and a 24-hours shift, and they showed frequent episodes of tachycardia reaching even up to 180 bpm and the cardiac stress was twice on the shift day compared to the non-shift days [9].

In patients who have already had previous cardiac events, these changes can put an extra strain and can worsen the prognosis of the disease. A study conducted in Japan by Kanno et al. examined the effects of insomnia on patients of heart failure and demonstrated that insomnia can worsen heart failure’s prognosis [10]. In their study, the patients with insomnia demonstrated significantly high rennin (p = 0.042) and aldosterone levels (p = 0.047) [10]. Similarly, in patients with ST-elevation myocardial infarction (STEMI) and insomnia, melatonin improved their sleep quality and reduced anxiety compared to oxazepam and was found to have a favorable effect on their cardiovascular health [12], SLEPT trial, on the other hand, showed that while sleep intervention improves the overall psychosocial health, it did not show much improvement in 24-hour, systolic blood pressure readings over a short duration [11]. On the other hand, sleep intervention therapies like cognitive behavioral therapy (CBT) or Tai Chi Chih (TCC) showed an improvement in cardiovascular risk after a year, as demonstrated in a study that measured the effects of sleep on biomarkers like lipid profile, inflammatory markers, and insulin [13].

## Conclusions

The circadian rhythm is responsible for a streamlined and coordinated functioning of different cardiovascular, metabolic, and immunological functions. These functions are controlled by cyclical peaks and troughs in the production and levels of different hormones and biological functions. Shift work and certain sleep disorders like insomnia, obstructive sleep apnea, and reduced sleep can cause a state of circadian misalignment, which increases the risk of developing cardiovascular diseases. This effect can be directly attributed to cortisol or melatonin levels or indirectly through its effects on metabolic and immunological functions. Shift work, if accompanied by stressful work conditions as faced by physicians and other healthcare professionals, can further contribute to increasing the risk of cardiovascular diseases. Therefore, we conclude that the risk of cardiovascular disorders can be reduced by treating the disorders causing circadian misalignment, and this option should always be considered while calculating the risk of cardiovascular diseases.

## References

[1] Benjamin EJ, Muntner P, Alonso A (2019). Heart disease and stroke statistics—2019 update: a report from the American Heart Association. Circulation.

[2] Morris CJ, Yang JN, Scheer FA (2012). The impact of the circadian timing system on cardiovascular and metabolic function. Prog Brain Res.

[3] Gooley JJ, Chamberlain K, Smith KA (2011). Exposure to room light before bedtime suppresses melatonin onset and shortens melatonin duration in humans. J Clin Endocrinol Metab.

[4] Scheer FA, Hilton MF, Mantzoros CS, Shea SA (2009). Adverse metabolic and cardiovascular consequences of circadian misalignment. Proc Natl Acad Sci U S A.

[5] Kervezee L, Kosmadopoulos A, Boivin DB (2018). Metabolic and cardiovascular consequences of shift work: the role of circadian disruption and sleep disturbances. Eur J Neurosci.

[6] Morris CJ, Purvis TE, Hu K, Scheer FA (2016). Circadian misalignment increases cardiovascular disease risk factors in humans. Proc Natl Acad Sci U S A.

[7] Barger LK, Rajaratnam SMW, Cannon CP (2020). Short sleep duration, obstructive sleep apnea, shiftwork, and the risk of adverse cardiovascular events in patients after an acute coronary syndrome. J Am Heart Assoc.

[8] Jarrin DC, Ivers H, Lamy M, Chen IY, Harvey AG, Morin CM (2018). Cardiovascular autonomic dysfunction in insomnia patients with objective short sleep duration. J Sleep Res.

[9] Dutheil F, Marhar F, Boudet G (2017). Maximal tachycardia and high cardiac strain during night shifts of emergency physicians. Int Arch Occup Environ Health.

[10] Kanno Y, Yoshihisa A, Watanabe S (2016). Prognostic significance of insomnia in heart failure. Circ J.

[11] McGrath ER, Espie CA, Power A (2017). Sleep to lower elevated blood pressure: a randomized controlled trial (SLEPT). Am J Hypertens.

[12] Ghaeli P, Solduzian M, Vejdani S, Talasaz AH (2018). Comparison of the effects of melatonin and oxazepam on anxiety levels and sleep quality in patients with ST-segment-elevation myocardial infarction following primary percutaneous coronary intervention: a randomized clinical trial. Ann Pharmacother.

[13] Carroll JE, Seeman TE, Olmstead R (2015). Improved sleep quality in older adults with insomnia reduces biomarkers of disease risk: pilot results from a randomized controlled comparative efficacy trial. Psychoneuroendocrinology.

[14] Dominguez-Rodriguez A, Abreu-Gonzalez P, Sanchez-Sanchez JJ, Kaski JC, Reiter RJ (2010). Melatonin and circadian biology in human cardiovascular disease. J Pineal Res.

[15] Tengattini S, Reiter RJ, Tan DX, Terron MP, Rodella LF, Rezzani R (2008). Cardiovascular diseases: protective effects of melatonin. J Pineal Res.

[16] Muller JE, Tofler GH (1991). Circadian variation and cardiovascular disease. N Engl J Med.

[17] Reilly DF, Westgate EJ, FitzGerald GA (2007). Peripheral circadian clocks in the vasculature. Arterioscler Thromb Vasc Biol.

[18] Scheer FA, Michelson AD, Frelinger AL 3rd (2020). The human endogenous circadian system causes greatest platelet activation during the biological morning independent of behaviors. PLoS One.

[19] Brezinski DA, Tofler GH, Muller JE (1988). Morning increase in platelet aggregability. Association with assumption of the upright posture. Circulation.

[20] Libby P (2012). Inflammation in atherosclerosis. Arterioscler Thromb Vasc Biol.

[21] Rajaratnam SM, Arendt J (2001). Health in a 24-h society. Lancet.

[22] Knutsson A (2003). Health disorders of shift workers. Occup Med (Lond).

[23] Dominguez-Rodriguez A, Abreu-Gonzalez P, Kaski JC (2009). Disruption of normal circadian rhythms and cardiovascular events. Heart Metab.

[24] Dominguez-Rodriguez A, Abreu-Gonzalez P, Reiter RJ (2020). Melatonin and cardiovascular disease: myth or reality?. Rev Esp Cardiol (Engl Ed).

[25] Thayer JF, Lane RD (2007). The role of vagal function in the risk for cardiovascular disease and mortality. Biol Psychol.

[26] von Känel R, Thayer JF, Fischer JE (2009). Nighttime vagal cardiac control and plasma fibrinogen levels in a population of working men and women. Ann Noninvasive Electrocardiol.

